# Systematic review and meta-analysis of pertussis epidemiology in Latin America and the Caribbean: 1980–2015

**DOI:** 10.26633/RPSP.2017.102

**Published:** 2017-11-17

**Authors:** Temitope Folaranmi, Veronica Pinell-McNamara, Matthew Griffith, Yongping Hao, Fatima Coronado, Elizabeth C. Briere

**Affiliations:** 1 Epidemic Intelligence Service Centers for Disease Control and Prevention AtlantaGeorgiaaddr United States of America Epidemic Intelligence Service, Centers for Disease Control and Prevention, Atlanta, Georgia, United States of America.; 2 Division of Bacterial Diseases National Center for Immunization and Respiratory Diseases, Centers for Disease Control and Prevention AtlantaGeorgia United States of America Division of Bacterial Diseases, National Center for Immunization and Respiratory Diseases, Centers for Disease Control and Prevention, Atlanta, Georgia, United States of America.; 3 Infectious Disease Surveillance Center National Institute of Infectious Diseases Tokyo Japan Infectious Disease Surveillance Center, National Institute of Infectious Diseases, Tokyo, Japan.; 4 Division of Scientific Education and Professional Development Center for Surveillance, Epidemiology and Laboratory Services, Centers for Disease Control and Prevention AtlantaGeorgia United States of America Division of Scientific Education and Professional Development, Center for Surveillance, Epidemiology and Laboratory Services, Centers for Disease Control and Prevention, Atlanta, Georgia, United States of America.

**Keywords:** Bordetella pertussis, pertussis vaccine, diphtheria-tetanus-pertussis vaccine, whooping cough, diphtheria-tetanus-acellular pertussis vaccines, Latin America, West Indies, Bordetella pertussis, vacuna contra la tos ferina, vacuna contra difteria, tétanos y tos ferina, tos ferina, vacunas contra difteria, tétanos y tos ferina acelular, América Latina, Indias Occidentales, Bordetella pertussis, vacina contra coqueluche, vacina contra difteria, tétano e coqueluche, coqueluche, vacinas contra difteria, tétano e coqueluche acelular, América Latina, Índias Ocidentais

## Abstract

**Objectives.:**

*In Latin America and the Caribbean (LAC), pertussis disease incidence has reportedly increased since 2000 despite high vaccine coverage. A systematic review of pertussis literature and a meta-analysis was conducted to understand the burden of disease in LAC*.

**Methods.:**

*A systematic literature review was completed, using relevant search terms. Original articles describing pertussis epidemiology and vaccine coverage in LAC published between 1980 and 2015 were identified. Applying a Bayesian meta-analysis random-effects model, we calculated pooled estimates and corresponding 95% credible intervals (95% CrIs) for pertussis incidence, case fatality ratio (CFR), pertussis prevalence among contacts, and coverage with three doses of diphtheria, tetanus, and pertussis (DTP) vaccine (DTP3)*.

**Results.:**

*A total of 59 studies meeting our selection criteria were identified, representing 15 countries. Of the 59, 15 of them provided incidence data, with 7 of the 15 reporting a pertussis case definition. The pertussis incidence estimate for the 1980–1999 period was 17.8 cases per 100 000 persons (95% CrI: 5.9–29.7); for the 2000–2015 period, it was 2.5 cases per 100 000 persons (95% CrI: 1.8–3.2). For the 1980–2015 period, the CFR, in 19 studies reviewed, was 3.9% (95% CrI: 2.9%–4.9%); for that same period, in 5 studies reviewed, pertussis prevalence among contacts was 24.9% (95% CrI: 13.7%–36.1%). Pooled DTP3 vaccine coverage estimates, in a total of 20 studies reviewed for the following three time periods, were: 1980–1990, 72.4% (95% CrI: 64.6%–80.2%); 1991–2000, 79.0% (95% CrI: 66.1%–91.9%); and 2001–2015, 90.0% (95% CrI: 87.7%–92.3%)*.

**Conclusion.:**

*A decrease in pertussis incidence and an achievement of moderately high DTP3 vaccine coverage since the early 2000s was observed. The review highlights the need for increased publication of pertussis data at the country level and for LAC as a whole in order to better understand the true burden of the disease. Application of a standardized case definition and use of active case finding would aid in obtaining more accurate estimates of the disease burden in LAC*.

## RESUMEN

**Objetivos.:**

En América Latina y el Caribe, la incidencia de la tos ferina habría aparentemente aumentado desde el 2000, a pesar de la alta cobertura de vacunación. Se realizó una revisión sistemática de la bibliografía sobre tos ferina y un metanálisis para conocer la carga de esta enfermedad en América Latina y el Caribe. Métodos. La revisión bibliográfica sistemática se realizó utilizando términos de búsqueda pertinentes. Se encontraron artículos originales que describían las características epidemiológicas de la tos ferina y la cobertura de vacunación en América Latina y el Caribe publicados entre 1980 y el 2015. Aplicando un modelo bayesiano de efectos aleatorios para el metanálisis, se obtuvieron estimaciones combinadas y los correspondientes intervalos de credibilidad del 95% (ICr) para la incidencia, la tasa de letalidad y la prevalencia entre contactos de la tos ferina, y la cobertura con tres dosis de vacuna contra la difteria, el tétanos y la tos ferina (DTP3).

**Resultados.:**

Se encontraron en total 59 estudios de 15 países que cumplían con los criterios de selección. De ellos, 15 proporcionaban datos sobre incidencia. Siete de estos 15 contenían una definición de caso de tos ferina. La incidencia estimada de tos ferina para el período 1980-1999 fue de 17,8 casos por 100 000 (ICr 95%: 5,9-29,7) y para el período 2000-2015, de 2,5 casos por 100 000 (ICr 95%: 1,8-3,2). En cuanto a la tasa de letalidad, para el período 1980-2015 en 19 estudios examinados fue de 3,9% (ICr 95%: 2,9%-4,9%); en el mismo período, en 5 estudios examinados la prevalencia de tos ferina entre los contactos fue de 24,9% (ICr 95%: 13,7%-36,1%). Las estimaciones combinadas de cobertura con DTP3 en un total de 21 estudios examinados para los siguientes tres períodos fueron: en 1980-1990, 72,4% (ICr 95%: 64,6%-80,2%); en 1991-2000, 79,0% (ICr 95%: 66,1%-91,9%) y en el 2001-2015, 90,0% (ICr 95%: 87,7%-92,3%).

**Conclusiones.:**

Se observó una disminución de la incidencia de la tos ferina y el logro de una cobertura moderadamente alta con la vacuna DTP3 desde principios del siglo XXI. En el examen se subraya la necesidad de incrementar la publicación de datos sobre la tos ferina en los países y en América Latina y el Caribe en su conjunto, para conocer mejor la verdadera carga de enfermedad. La adopción de una definición de caso normalizada y la búsqueda activa de casos ayudarán a obtener estimaciones más precisas de la carga de enfermedad en América Latina y el Caribe.

## RESUMO

**Objetivos.:**

Há relatos de que a incidência de coqueluche na América Latina e Caribe (ALC) tem aumentado desde o ano 2000, apesar da alta cobertura vacinal. Realizamos uma revisão sistemática e metanálise da literatura sobre a coqueluche para compreender a carga da doença na ALC. Métodos. Fizemos uma revisão sistemática da literatura usando termos de pesquisa relevantes. Identificamos artigos originais, publicados entre 1980 e 2015, que descrevessem a epidemiologia da coqueluche e a cobertura vacinal na ALC. Aplicando um modelo Bayesiano de efeitos aleatórios para a metanálise, calculamos estimativas agrupadas e seus correspondentes intervalos de credibilidade de 95% (CrIs 95%) para a incidência de coqueluche, a taxa de letalidade, a prevalência de coqueluche entre os contatos e a cobertura com três doses da vacina combinada para difteria, tétano e coqueluche (DTP3).

**Resultados.:**

Identificamos um total de 59 estudos que cumpriram os nossos critérios de seleção, representando 15 países. Destes 59, 15 apresentaram dados sobre a incidência, e 7 dos 15 apresentaram uma definição de “caso de coqueluche”. A incidência estimada da coqueluche no período de 1980 a 1999 foi de 17,8 casos por 100.000 pessoas (CrI 95%: 5,9-29,7); no período de 2000 a 2015, foi de 2,5 casos por 100.000 pessoas (CrI 95%: 1,8-3,2). No período de 1980 a 2015, a taxa de letalidade, em 19 estudos revistos, foi de 3,9% (CrI 95%: 2,9%-4,9%); neste mesmo período, em 5 estudos revistos, a prevalência de coqueluche entre os contatos foi de 24,9% (CrI 95%: 13,7%-36,1%). A cobertura vacinal agrupada com DTP3, em um total de 21 estudos examinados nos seguintes três períodos, foi estimada em: 1980 a 1990, 72,4% (CrI 95%: 64,6%-80,2%); 1991 a 2000, 79,0% (CrI 95%: 66,1%-91,9%); 2001 a 2015, 90,0% (CrI 95%: 87,7%-92,3%).

**Conclusões.:**

Foi observada uma redução na incidência de coqueluche e uma cobertura vacinal com DTP3 relativamente alta desde o início da década de 2000. Esta revisão destaca a necessidade de melhorar a publicação de dados sobre a coqueluche ao nível nacional e na ALC como um todo, a fim de promover uma melhor compreensão sobre a verdadeira carga da doença. O uso de uma definição padronizada de “caso de coqueluche” e a busca ativa de casos ajudaria na obtenção de estimativas mais precisas da carga da doença na ALC.

Globally, pertussis is a poorly controlled disease that causes substantial morbidity and mortality. An estimated 16 million cases of the disease and approximately 195 000 deaths occur worldwide every year (1). After introduction of diphtheria, tetanus, and pertussis (DTP) vaccines during the early 1980s and reported vaccination coverage with three doses of DTP (DTP3) above 90% by the late 1990s, the incidence of pertussis had decreased substantially in Latin America and the Caribbean (LAC) (2–6). More recently, however, pertussis disease incidence has reportedly increased since 2000 (7–10). For example, in Argentina, Brazil, and Colombia, the number of reported cases during 2013 increased by 116%, 582%, and 2 967%, respectively, compared with cases reported during 2000 (11), and outbreaks have been reported in multiple countries during the last decade (6, 12–15). In most LAC countries, pertussis disease is nationally notifiable through a passive surveillance system; however, variation exists among surveillance systems, making comparability of data in LAC a challenge (7).

We conducted a systematic review of literature on pertussis in LAC that was published between 1980 and 2015, in order to more fully understand the burden of disease and vaccine coverage in LAC and to provide historical context as certain LAC countries consider a transition from a whole-cell (wP) to an acellular (aP) primary vaccine series.

## METHODS

We conducted a systematic review and meta-analysis of original articles meeting our selection criteria. We systematically searched the following electronic databases: MEDLINE, EMBASE, Cumulative Index to Nursing and Allied Health Literature (CINAHL), Web of Science, Cochrane Library, Latin American and Caribbean Health Sciences Literature (LILACS), Pan American Health Organization (PAHO) Virtual Health Library, World Health Organization Library Database (WHOLIS), Scientific Electronic Library Online (SciELO), and CAB Direct and Global Health. For the search, we used the following disease search terms specifically focused on LAC: whooping cough OR pertus* OR whooping cough OR tos ferina OR tosferina OR coqueluche.

### Eligibility criteria

We sought original articles describing pertussis epidemiology and pertussis vaccination coverage published in English, French, Spanish, or Portuguese from January 1980 through December 2015 for all populations in South America, Central America, Mexico, and the Caribbean. To be included, articles were required to describe studies or surveillance that focus on humans as subjects; contain greater than 30 index cases in studies reporting prevalence among contacts; focus on the general population; and be published in a peer-reviewed journal (an exception was made for government reports found through a systematic electronic database review). We excluded review articles, outbreak reports, case reports, position papers, reports concerning special populations (e.g., prisoners), and dissertations. A library science expert assisted with the search. Appendix 1 gives additional details on the search strategy.

### Study selection

Three reviewers independently screened all titles and abstracts that the search yielded. Results were reconciled, and irrelevant and duplicate citations were excluded. The reviewers independently reviewed the full text of the remaining available articles to judge their eligibility for inclusion in the review. For all included articles, we abstracted information regarding report characteristics, sample selection methods, sample characteristics, sample participation rates, report findings (for the principal outcomes), and relevant *P* values or confidence intervals. When denominators were not given, we back-calculated for the denominator using the numerator and the outcome estimates provided. The data were entered into a database using Microsoft Excel software (Microsoft Corporation, Redmond, Washington, United States of America).

### Outcomes measures

The principal outcomes included were: pertussis incidence in the general population of all age groups (defined as confirmed cases of pertussis/100 000 persons); pertussis prevalence among contacts (defined as laboratory-confirmed, symptomatic cases/100 evaluated contacts); pertussis case fatality ratio (CFR) (defined as number of deaths/100 confirmed pertussis cases); DPT3 vaccination coverage (defined as the number of subjects aged 6 months through 5 years vaccinated with three DPT vaccine doses/100 eligible subjects assessed); pertussis laboratory confirmation (defined as pertussis cases confirmed with a specific laboratory technique); and pertussis hospitalization (average duration of days hospitalized for a laboratory-confirmed case of pertussis). DPT3 coverage is a widely accepted indicator for assessing routine immunization coverage, and it is frequently used as a proxy for assessing the quality of immunization systems.

### Analysis

Applying a Bayesian meta-analysis random-effects model, we calculated pooled estimates and corresponding 95% credible intervals (CrIs), with 20 articles describing pertussis vaccination coverage and with 15 articles describing pertussis incidence rates. A random-effects model assumes the studies are a sample from all possible studies. Thus, this approach takes into account variance both within each study and between studies (16). If an outcome had insufficient data points available for statistical pooling, a descriptive summary of the data was presented instead.

Pertussis vaccination coverage data were stratified into three time periods (1980–1990; 1991–2000; and 2001–2015), and pertussis incidence data were stratified into two time periods (1980–2000 and 2001–2015). In addition to plotting the individual study estimates and CrIs, we calculated pooled random-effects estimates and 95% CrIs for each category. Stratification of the data into the above time periods is based on articles describing data clustered around specific time frames. For vaccination coverage data, we also conducted Bayesian hierarchical logistic regression to determine trend over time. All analyses were implemented by using the SAS PROC MCMC (Markov chain Monte Carlo) procedure. SAS version 9.3 (SAS Institute Incorporated, Cary, North Carolina, United States) was used for the analysis.

## RESULTS

**FIGURE 1.**
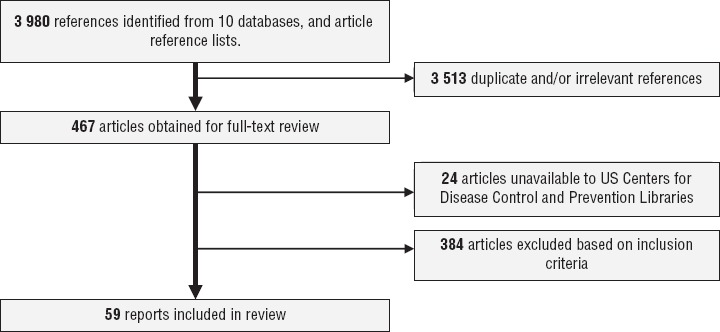
Literature search strategy and selection process

***Source:*** Produced by authors from the results of the analysis.

Of the 3 980 references identified, 3 513 were duplicates or irrelevant publications, 24 were unavailable to the Centers for Disease Control and Prevention (CDC) library, and 384 were excluded based on inclusion criteria, leaving 59 articles for review (8, 17–74) (Figure 1), representing 15 countries (Table 1). The largest numbers of the included articles were from Brazil (32.2%), Argentina (15.2%), and Colombia (11.9%). Twenty articles (32.2%) reported findings concerning vaccination coverage (20, 21, 24, 26, 27, 30, 35–37, 39, 43, 44, 48, 50, 52, 53, 56–59) (Appendix 2), and 40 (67.7%) reported findings concerning pertussis epidemiology (8, 17–19, 22, 23, 25, 28–34, 38, 40–42, 45–47, 49, 51, 54, 55, 60–74) (Appendix 3). The largest numbers of the articles were published in either Spanish (45.7%) or English (32.2%) (Table 1).

**TABLE 1.** Characteristics of studies included in the analysis

Publication Characteristics	N	%	References
Language			
Spanish	27	46	(21–25, 28, 30, 33, 34, 36, 38, 40–43, 45, 49, 51, 52, 54–56, 61, 62, 68, 72, 73)
Portuguese	12	20	(19, 27, 35, 39, 44, 47, 50, 57–60, 71)
English	19	32	(8, 17, 18, 20, 26, 29, 31, 32, 46, 48, 53, 63–67, 69, 70, 74)
French	1	2	(37)
Country	**59**	**100**	
Argentina	9	15	(8, 26, 31, 32, 34, 36, 40, 55, 70)
Brazil	19	32	(17–19, 27, 29, 35, 39, 44, 47, 50, 57–60, 63, 66, 69, 71, 74)
Chile	3	5	(28, 30, 49)
Colombia	7	12	(24, 41–43, 54, 56, 73)
Costa Rica	2	3	(21, 33)
Ecuador	1	2	(20)
Guyana	1	2	(48)
Haiti	1	2	(53)
Honduras	2	3	(38, 52)
Martinique	1	2	(37)
Mexico	5	8	(22, 23, 46, 61, 68)
Panamá	1	2	(45)
Peru	3	5	(64, 65, 67)
Uruguay	3	5	(51, 62, 72)
Venezuela	1	2	(25)

***Source:*** Produced by authors from analysis results.

### Pertussis epidemiology

**FIGURE 2.**
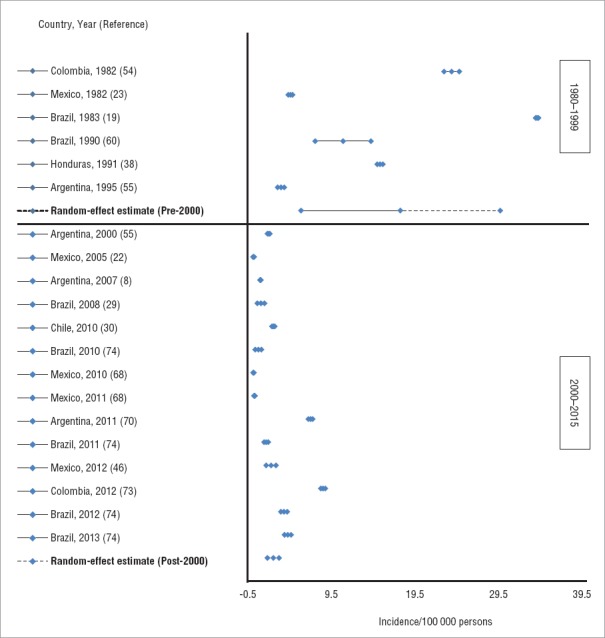
Random-effect estimate of pertussis incidence, 1980–2015

***Source:*** Produced by authors from the results of the analysis.

***Note:*** Random-effect estimate is a pooled estimate which allows for variations in the treatment effect from study to study. The effects in individual studies are assumed to be random realizations of the “true” effect. The data being analyzed in individual studies are then assumed to be drawn from populations with the randomly realized effects.

Forty publications reported pertussis epidemiologic data from 11 countries: Argentina, Brazil, Chile, Colombia, Costa Rica, Honduras, Mexico, Panama, Peru, Uruguay, and Venezuela (Appendix 3). Fifteen publications reported pertussis incidence outcome data (8, 19, 22, 23, 29, 30, 38, 46, 54, 55, 60, 68, 70, 73, 74). Two publications (55, 68) reported data for two years each (respectively, for 1995 and 2000, and for 2010 and 2011). Another publication (74) reported data for four years, for 2010 through 2013. Although 8 of these 15 articles (53.3%) (8, 19, 29, 30, 46, 55, 72, 74) reported a case definition, only 6 (40.0%) used the WHO pertussis case definition (75). In addition, variation among the case definitions in the remaining articles was noted. For example, the number and type of presenting symptoms and the duration of cough required to meet the case definition differed in each study. Seven of the 8 articles (87.5%) included laboratory confirmation as part of their case definition. Two articles (30, 68) did not specify the type of diagnostic used for laboratory confirmation; in the remaining 5 articles, 1 included only culture for confirmation (29), 3 included both culture and polymerase chain reaction (PCR) (46, 55, 74), and 1 included culture, PCR, and serology (8). By applying a random-effects model that included the 15 publications reporting incidence data, the pooled estimates of annual pertussis incidence in the general population during 1980–1999 and 2000–2015 were, respectively, 17.8 cases per 100 000 persons (95% CrI: 5.9–29.7) and 2.5 cases per 100 000 persons (95% CrI: 1.8–3.2) (Figure 2).

**FIGURE 3.**
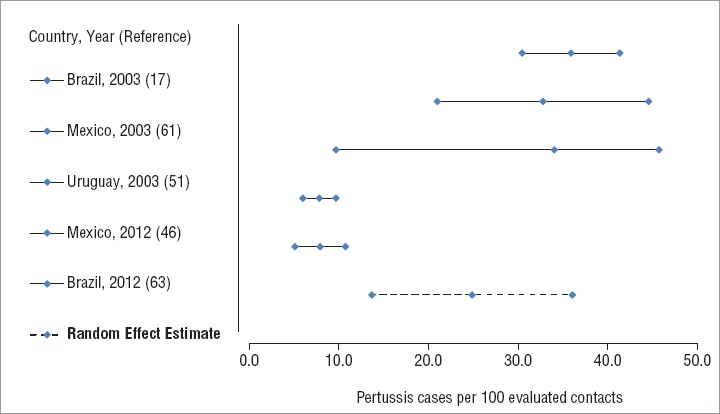
Random-effect estimate of pertussis prevalence among contacts of confirmed cases, 1980–2015

***Source:*** Produced by authors from the results of the analysis

***Note:*** Random effect estimate is a pooled estimate which allows for variations in the treatment effect from study to study. The effects in individual studies are assumed to be random realization of the “true” effect. The data being analyzed in individual studies are then assumed to be drawn from populations with the randomly realized effects.

The prevalence of symptomatic pertussis among contacts of laboratory-confirmed pertussis cases was reported in five publications (17, 46, 51, 61, 63). All five focused on pertussis among infants or school children, but they reported symptomatic pertussis among all close contacts, using epidemiologic link or laboratory confirmation with PCR, culture, or serology. Among contacts of confirmed cases evaluated, an estimated 24.9% (95% CrI: 13.7%–36.1%) had laboratory-confirmed, symptomatic pertussis (Figure 3). None of the studies reported whether the contacts had received chemoprophylaxis before symptom onset, or the length of time from case symptom onset to contact specimen collection.

**FIGURE 4.**
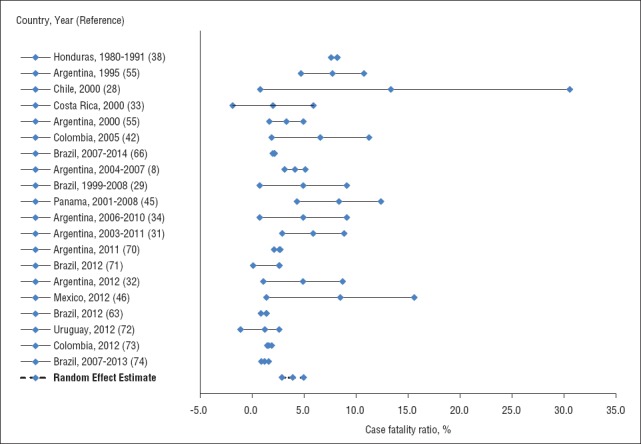
Random-effect estimate of the fatality ratio in cases of pertussis in Latin America, 1980–2015

***Source:*** Produced by authors from the results of the analysis

***Note:*** Random effect estimate is a pooled estimate which allows for variations in the treatment effect from study to study. The effects in individual studies are assumed to be random realizations of the “true” effect. The data being analyzed in individual studies are then assumed to be drawn from populations with the randomly realized effects.

Nineteen publications reported mortality data (8, 28, 29, 31–34, 38, 42, 45, 46, 55, 63, 66, 70-74). CFR was estimated to be 3.9% (95% CrI: 2.9%–4.9%) (Figure 4). Of the 19 publications, 7 of them (36.8%) were hospital-based studies focusing on infants and school-age children, whereas 12 (63.2%) used surveillance data covering infants, adolescents, and adults.

Twenty-four articles reported laboratory confirmation data (18, 22, 25, 28, 29, 31–34, 40, 41, 45, 47, 49, 51, 61–65, 67, 69, 71, 73). Laboratory confirmation using culture, PCR, and/or direct fluorescent antibody (DFA) testing alone was reported, respectively, in 13 (54.2%), 14 (58.3%), and 3 (12.5%) publications. Of the 24 articles with laboratory confirmation data, 7 studies (29.2%) used PCR and culture as complimentary diagnostic tests (40, 51, 62, 63, 65, 67, 69). Use of PCR as a laboratory confirmation method was reported as early as 1995 in 1 article (49), but it became more common over time, with the majority of publications published after 2006 reporting PCR for pertussis confirmation.

Eight publications, from four countries (Argentina, Brazil, Panama, and Uruguay), reported hospital duration data among patients with confirmed pertussis (31, 32, 34, 40, 45, 51, 71, 72). Six publications reported mean hospital duration, ranging from 5.2 to 14.2 days. Two publications reported a median hospital duration, one of 7.0 days and the other of 9.7 days.

### Vaccination coverage data

Pertussis vaccination coverage data were reported in 20 publications (20, 21, 24, 26, 27, 30, 35–37, 39, 43, 44, 48, 50, 52, 53, 56–59), covering 10 countries. The 20 reports included 8 (40.0%) from Brazil, 3 (15.0%) from Colombia, 2 (10.0%) from Argentina, and 1 each from Chile, Costa Rica, Ecuador, Guyana, Haiti, Honduras, and Martinique (Appendix 2). All publications reported coverage with whole cell vaccines (DTP), because acellular vaccines were not used by the countries at the time of the coverage assessment. Sixteen publications (80.0%) estimated vaccination coverage data by using household surveys (20, 24, 26, 27, 35–37, 39, 43, 44, 50, 52, 53, 56–58), 3 publications (15.0%) (30, 48, 59) used the national immunization program data to estimate vaccination coverage, and 1 publication (5.0%) used a school-based survey (21). In 13 of the 20 publications (65.0%), survey samples were selected through systematic or random selection of participants, whereas in 7 studies (35.0%) the entire eligible population was surveyed. The age of the eligible population varied among the publications; however, 65.0% reported an eligible population less than age 5 years. Vaccination status was confirmed by vaccination cards or any written proof of vaccination in 10 of the publications (50.0%), whereas in 8 publications (40.0%), verbal report by guardians or parents was accepted if vaccination cards were unavailable.

**FIGURE 5.**
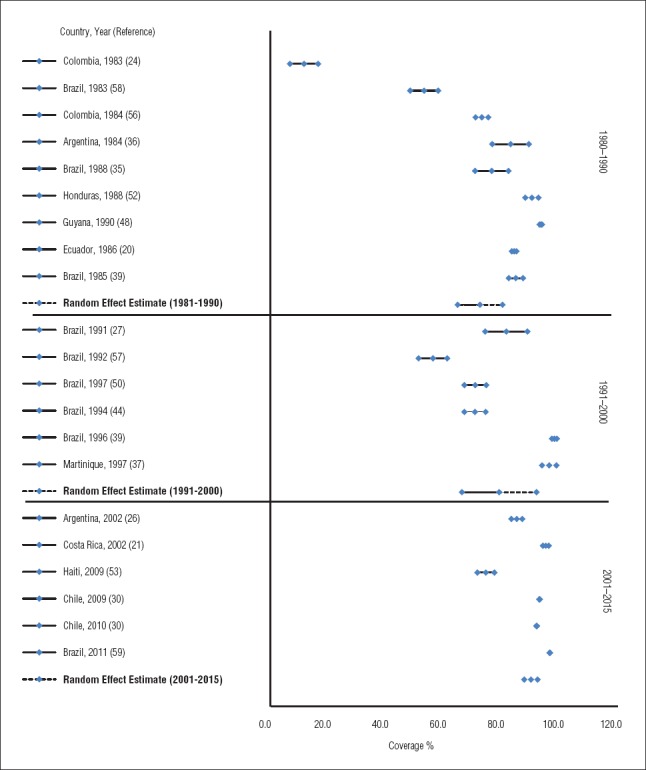
Random-effect estimate of DTP3 Vaccine coverage in Latin America, 1980–2015

***Source:*** Produced by authors from the results of the analysis

***Note:*** Random effect estimate is a pooled estimate which allows for variations in the treatment effect from study to study. The effects in individual studies are assumed to be random realization of the “true” effect. The data being analyzed in individual studies are then assumed to be drawn from populations with the randomly realized effects.

**FIGURE 6.**
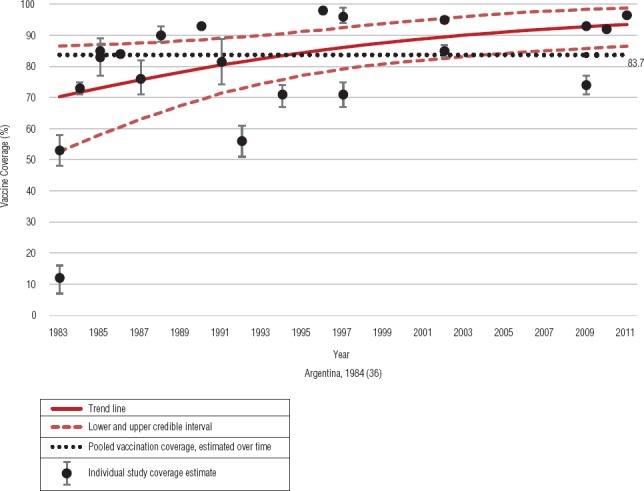
Trends in Vaccine Coverage, 1980–2015

***Source:*** Produced by authors from the results of the analysis

***Note:*** The big black dots represent estimated annual vaccine coverage from individual studies and the 95% Crls bands are illustrated along the vertical axis. The black dotted line represents the overall estimate of vaccine coverage during the study period. The bold red line represents the line of the best fit for vaccine coverage trend over time; the red dashed lines represent the upper and lower 95 Crl limit of the vaccine coverage trend.

DPT3 coverage data were assessed over three time periods, depending on the year the data were collected, as follows: 1980–1990 (20, 24, 35, 36, 39, 48, 52, 56, 58); 1991–2000 (27, 37, 39, 44, 50, 57); and 2001–2015 (21, 26, 30, 53, 59). Pooled estimates of DTP3 vaccination coverage over the time periods of 1980–1990, 1991–2000, and 2001–2015 were, respectively: 72.4% (95% CrI: 64.6%–80.2%), 79.0% (95% CrI: 66.1%–91.9%), and 90.0% (95% CrI: 87.7%–92.3%) (Figure 5). The overall pooled estimate for the entire 1980–2015 time period is 83.7% (95% CrI: 75.1%–91.4%). Furthermore, using a Bayesian hierarchical logistic regression model, we estimated an overall increase in vaccination coverage over time, as shown by the solid red line (with upper and lower CrIs indicated by the dashed red lines) in Figure 6.

## DISCUSSION

This is the first systematic review of pertussis epidemiology and vaccine coverage in Latin America and the Caribbean, thus contributing to a better understanding of pertussis disease in LAC countries. From our analysis we observed a clear decrease in pertussis incidence during 1980-1999 and 2000–2014, as well as achievement of moderately high DTP3 vaccination coverage since the early 2000s.

The decline in pertussis incidence observed between 1980 and 2000 is consistent with WHO data and likely attributable to the widespread introduction of pertussis vaccines during the 1980s. Because of the limited data available, we were unable to stratify pertussis incidence data by finer time periods and, for example, assess whether any increase in pertussis incidence occurred within the last decade. However, the WHO Strategic Advisory Group of Experts (SAGE) pertussis working group recently reviewed pertussis surveillance data from 19 countries, including 4 from LAC (Brazil, Chile, Cuba, and Mexico), and determined there has been no true resurgence of pertussis in these 4 LAC countries. Instead, increases were attributable to naturally occurring cyclic patterns, surveillance systems changes, or decreases in vaccination coverage (76, 77). It is also important to note that despite recent increases in reported pertussis cases in LAC, the majority of countries are not reporting case counts as high as those reported during the 1980s (11).

Our review highlighted the complexities of comparing pertussis incidence among countries. For example, case definitions for pertussis varied among publications. Consequently, there might have been under-or overestimates of the burden of disease. Among those publications with a case definition, the definitions were inconsistent, and fewer than half of them used the WHO pertussis case definition (75). Creating a standardized pertussis case definition is complicated because of variations in clinical signs and symptoms of pertussis among different age groups (78, 79). However, a standardized, age-specific clinical case definition for LAC would make it easier to compare data between countries in LAC and to estimate the true burden of pertussis there (79).

Similarly, we note the challenges associated with using diagnostic methods with different levels of sensitivity and specificity. Although culture is considered the gold standard for pertussis diagnosis, it has a relatively low sensitivity. Therefore, CDC encourages using complementary diagnostic methods (combination of PCR, culture, and serology) to strengthen a surveillance system’s potential for accurate case confirmation. Of the 24 articles that we reviewed that had laboratory confirmation data, only 14 (58.3%) used culture as a diagnostic method, and only 7 (29.2%) used PCR and culture as complementary diagnostic methods. DFA is not recommended by WHO and CDC as a pertussis laboratory diagnostic because of low sensitivity and specificity (80–82); nevertheless, certain countries continue to use it as a diagnostic method. The majority of the studies we reviewed for laboratory confirmation methods were hospital based; therefore, their diagnostic methods might not be the same as those used in the national surveillance system. Efforts to standardize specimen collection, transportation, and diagnostic techniques and to establish continuous quality control and quality assurance programs will increase case confirmation and improve comparability of laboratory data within and across LAC countries.

Our review indicated an increase in DTP3 vaccination coverage over the three time periods. Our modeled analysis reported an increase in vaccination coverage over time although we observed an overlap in credible intervals among pooled estimates for the three time periods. The majority of the studies included in our review were household surveys and might provide a more accurate estimate of coverage in LAC than administrative data, which often overestimate vaccination coverage. Our 2001–2015 DTP3 coverage estimate met the WHO Global Immunization Vision and Strategy (GIVS) global target of ≥ 90% vaccination coverage (83), but did not meet the PAHO Technical Advisory Group on Vaccine-preventable Diseases target of ≥ 95% coverage among children aged < 1 year (9). To ensure high protection levels among children aged < 5 years and prevent increases in disease burden, LAC countries still need to make efforts to meet and maintain these targets.

The CFR of 3.9% estimated in our analysis was consistent with other estimates of pertussis CFR among lower-income countries (77) but much higher than the United States estimates of 0.15% during a similar time period. The high CFR in our analysis may be an overestimate due to reporting bias if the countries’ surveillance systems were not sensitive enough to identify less severe cases of pertussis. Although we were unable to calculate CFR by different age groups in our review, the majority of reported cases in LAC are infants, and thus this CFR likely represents infant mortality. Infants are at highest risk for severe pertussis disease and complications; therefore, rapid access to quality tertiary care is critical to prevent infant deaths. The LAC CFR might be a reflection of different levels of health care access and quality, especially in rural areas. A high level of suspicion among health care providers and early diagnosis and treatment are important for reducing pertussis mortality.

We estimated a pertussis prevalence rate of 24.9% among close contacts of confirmed pertussis cases. Secondary attack rates among susceptible household contacts of confirmed cases as high as 80% to 90% have been reported in previous studies (84, 85). Early contact investigation to ensure that contacts of confirmed cases receive prophylaxis is key in preventing secondary transmission, especially among households with persons at high risk (e.g., pregnant women, infants, and the immunocompromised) (86). In multiple studies, siblings are frequently the source of infant infection; therefore, vaccination of siblings to prevent primary infection and subsequent household transmission is important (17, 87, 88).

Our review can provide baseline data to evaluate the impact of vaccination changes in LAC on pertussis disease burden. Certain LAC countries are considering a transition from a whole-cell (wP) to an acellular (aP) primary vaccine series. Countries that have already introduced acellular vaccines for the infant doses include Costa Rica and Mexico (7), as well as Panama (personal communication with the pertussis surveillance coordinator of the Panama Ministry of Health). However, data from multiple countries outside Latin America that use an aP primary vaccine series (77, 89–95) indicate the duration of protection offered by aP vaccines wanes over time. In addition, data from modeling studies indicate the transition from wP to aP vaccines might result in a resurgence of disease (96–98). Therefore, a switch to an aP vaccine series at this time might potentiate further increases in pertussis incidence in Latin America. On the basis of available evidence, PAHO’s Technical Advisory Group on Vaccine-preventable Diseases recommends that countries currently using wP vaccines should not switch to aP vaccines (9).

The introduction of tetanus-diphtheria-acellular pertussis (Tdap) booster vaccines for pregnant women in certain Latin American countries (Argentina, Brazil, Chile, Colombia, Costa Rica, Mexico, and Panama) might affect pertussis incidence by decreasing the disease burden among infants too young to be vaccinated (99–102). Data from the United Kingdom estimate high maternal vaccination effectiveness (103, 104). There is currently no PAHO recommendation for routine vaccination of pregnant women. However, the WHO acknowledges that maternal vaccination is likely the most cost-effective strategy for reducing infant disease. WHO therefore suggests that countries experiencing high rates of infant morbidity or mortality from pertussis consider use of maternal vaccination in addition to routine infant vaccination (77).

### Limitations

Our results may have been influenced by several limitations. To ensure our review included high-quality data that accurately represented the epidemiology of pertussis in LAC, we applied strict eligibility criteria to our search. However the number of articles meeting our eligibility criteria was limited, and only Argentina, Brazil, Chile, Colombia, Honduras, and Mexico were represented in our review of incidence data. Such paucity and geographic underrepresentation of data meeting our inclusion criteria limit the generalizability of our findings and may have led to over-or underestimates of pertussis incidence. In addition, the limited data prevented us from assessing changes in pertussis incidence within the last decade. Similarly, the heterogeneity of study methods and pertussis case definitions among the eligible articles limited data comparability and generalizability.

### Conclusions

Our review highlights the challenge of estimating the overall burden of pertussis in an area with substantial variation in surveillance systems, health care access, and vaccination coverage, both within and across countries. Our findings underline the need for increased publication of pertussis epidemiology data at a country level and within LAC as a whole. Such publications could provide a better understanding of the true burden of pertussis disease in LAC, especially as more countries implement maternal Tdap vaccination and consider transition to an acellular primary series. Use of a standardized case definition, laboratory confirmation with complementary diagnostic methods, and active case finding among contacts of confirmed pertussis cases would also aid in obtaining a more accurate estimate of pertussis disease burden in LAC.

#### Acknowledgments.

We thank Nong Shang for his statistical advice.

#### Disclaimer.

Authors hold sole responsibility for the views expressed in the manuscript, which may not necessarily represent the official position or policy of the Centers for Disease Control and Prevention, the *RPSP/PAJPH*, or PAHO.

## References

[1] 1. Black R, Cousens S, Johnson H, Lawn J, Rudan I, Bassani D, et al. Global, regional, and national causes of child mortality in 2008: a systematic analysis. Lancet. 2010;375(9730):1969–87. 10.1016/S0140-6736(10)60549-1 20466419

[2] 2. McCormick CM, Czachor JS. Pertussis infections and vaccinations in Bolivia, Brazil and Mexico from 1980 to 2009. Travel Med Infect Dis. 2013;11:146–51. 10.1016/j.tmaid.2013.04.002 23623448

[3] 3. Pan American Health Organization. Vaccinate your Family. Protect your Community: XIX Technical Advisory Group Meeting-Final Report 2011. Available from: http://www.paho.org/immunization/toolkit/resources/tech-recommendations/TAG-2011.pdf . Accessed in April, 2016.

[4] 4. Pan American Health Organization. Vaccine Coverage by Vaccine: DPT3 2014. Available from: http://ais.paho.org/phip/viz/im_coveragebyvaccine.asp. Accessed in April, 2016.

[5] 5. World Health Organization. Immunization Surveillance, Assessment and Monitoring: Diphtheria tetanus toxoid and pertussis (DTP3) immunization coverage among 1-year-olds, 1980–2014 (%) 2014 [Available from: http://gamapserver.who.int/gho/interactive_charts/immunization/dpt3/atlas.html?filter=filter4,Americas. Accessed in April, 2016

[6] 6. Pan American Health Organzation. Pertussis in the Americas-2007 [Internet]. Available from: http://www.paho.org/english/ad/fch/im/sne2906.pdf. Accessed in April, 2016

[7] 7. Arlant LHF, de Colsa A, Flores D, Brea J, Aguero MLA, Hozbor DF. Pertussis in Latin America: epidemiology and control strategies. Expert Rev Anti Infect Ther. 2014;12(10):1265–75. 10.1586/14787210.2014.948846 25139010

[8] 8. Hozbor D, Mooi F, Flores D, Weltman G, Bottero D, Fossati S, et al. Pertussis epidemiology in Argentina: trends over 2004–2007. J Infect. 2009;59(4):225–31. 10.1016/j.jinf.2009.07.014 19651156

[9] 9. Pan American Health Organzation. Meeting Report of Techincal Advisory Group in Vaccine-preventable Diseases (Oct, 2012)2012. Available from: http://www.paho.org/immunization/toolkit/resources/tech-recommendations/TAG-2012.pdf. Accessed in May, 2016.

[10] 10. Debbag R, Sarti E, Espinal C, Mascareñas C. Current pertussis epidemiological situation in Latin America and associated vaccination strategies. 31st annual meeting of the European Society for Paediatric Infectious Diseases; June 2013; Milan, Italy.

[11] 11. Pan American Health Organzation. Number of Vaccine Preventable Disease (VPD) cases in the Americas: Pertussis [Internet]. PAHO Immunization Unit 2015. Available from: http://ais.paho.org/phip/viz/im_vaccinepreventablediseases.asp. Accessed in May, 2016

[12] 12. Villareal C, Buelvas, D., Morón Duarte, L., Gomez, E., Castillo, O. Brote de tosferina, municipio de sincelejo departamento de sucre, Colombia, 2008. Investig andin. 2008;10(17):10.

[13] 13. Barrios de Leon E. Brote sospechoso de tos ferina en la Colonia Nueva, de la aldea El Amparo, Municipio de el Tumbador, San Marcos, Agoston 2004: Field Epidemiolog Training Program (FETP); 2004 [Available from: http://cedoc.cies.edu.ni/digitaliza/t293/secciona5.pdf. Accessed in May, 2016

[14] 14. Nieto Guevara J, Luciani K, Montesdeoca Melian A, Mateos Duran M, Estripeaut D, Nieto Guevara J, et al. Hospital admissions due to whooping cough: experience of the del niño hospital in Panama. Period 2001–2008. Anales de Pediatria. 2010;72(3):172–8. 10.1016/j.anpedi.2009.11.012 20153272

[15] 15. Ulloa-Gutierrez R, Avila-Aguero ML. Pertussis in Latin America: current situation and future vaccination challenges. Expert Rev Vaccines. 2008;7(10):1569–80. 10.1586/14760584.7.10.1569 19053212

[16] 16. Smith T, Spiegelhalter D, Thomas A. Bayesian Approaches to Random-Effects Meta-Analysis: A comparative Study. Stat Med. 1995;14(24):2685–99. 10.1002/sim.4780142408 8619108

[17] 17. Baptista PN, Magalhaes VS, Rodrigues LC. Children with pertussis inform the investigation of other pertussis cases among contacts. BMC Pediatr. 2007;7:21. 10.1186/1471-2431-7-21 PMC1894795 17518997

[18] 18. Baptista PN, Magalhães VS, Rodrigues LC. The role of adults in household outbreaks of pertussis. Int J Infect Dis. 2010;14(2):e111–4. 10.1016/j.ijid.2009.03.026 19559636

[19] 19. Brasil. Ministério da Saúde. Divisäo de S. Vigilância epidemiológica da coqueluche. Bol epidemiol (Rio J) 1985;17(9/10):69–72.

[20] 20. Brüssow H, Sidoti J, Freire WB. Tetanus and diphtheria immunization coverage in Ecuadorian children after a national vaccination campaign. J Infect Dis. 1993;168(2):479–83. 10.1093/infdis/168.2.479 8335991

[21] 21. Calvo N, Morice A, Sáenz E, Navas L. Uso de encuestas en escolares para la evaluación de la cobertura y oportunidad de la vacunación en Costa Rica. Rev Panam Salud Publica. 2004;16(2):118–24. 10.1590/s1020-49892004000800007 15357937

[22] 22. Capuchino Monreal Y, Salazar Montes L, Cárdenas Romero C. Perfil clínico-epidemiológico de la tos ferina en Jalisco en el periodo comprendido entre enero del año 2004 a septiembre del 2005. Boletin Mensual Guadalajara 2005.

[23] 23. Carrada Bravo T. Vigilancia epidemiológica de enfermedades transmisibles en niños. Rev mex pediatr. 1985;52(9):421–5.

[24] 24. Castillo A, Munoz M, Aguirre R. Aplicacion de normas epidemiologicas: tosferina en Frontino. Bol epidemiol Antioq. 1983;8(2):66–7.

[25] 25. David A, Kouris E, Álvarez M, Márquez MT, Martín A. Bordetella pertussis: caracterización epidemiológica y diagnóstico. Arch venez pueric pediatr. 2005;68(4):164–9.

[26] 26. Dayan GH. Vaccination coverage among children aged 13 to 59 months in Buenos Aires, Argentina, 2002. Rev Panam Salud Publica. 2004;16(3):158–67. 10.1590/s1020-49892004000900002 15507183

[27] 27. de Miranda AS, Scheibel IM, Tavares MRG, Takeda SMP. Avaliaçäo da cobertura vacinal do esquema básico para o primeiro ano de vida. Rev Saúde Públ 1995;29(3):208–14. 10.1590/s0034-89101995000300008 8539532

[28] 28. Donoso F. A, Wegner A. A, León B. J, Ramírez A. M, Carrasco O. JA. Coqueluche en niños menores de seis meses de vida. Rev Chil Pediat. 2001;72(4):334–9.

[29] 29. Druzian AF, Brustoloni YM, Oliveira SM, Matos VT, Negri AC, Pinto CS, et al. Pertussis in the central-west region of Brazil: one decade study. Braz J Infect Dis. 2014;18(2):177–80. 10.1016/j.bjid.2013.08.006 PMC9427500 24275370

[30] 30. Galleguillos B, Rubilar P. Tos ferina: una enfermedad que resurge en Chile. Vigía (Santiago).13(27):46–9.

[31] 31. Gentile Á, Romanin VS, Juárez MdV, Lución MF, Marques MdlÁ, Mistchenko AS. Epidemiología de Bordetella pertussis en un hospital pediátrico. Arch argent pediatr. 2014;112(1):26–32. 10.5546/aap.2014.eng.26 24566778

[32] 32. Gentile A, Salgueiro AL, Garcia Bournissen F, Romanin V, Bulgheroni S, Gaiano A, et al. Cost of Bordetella pertussis illness in tertiary hospitals in Argentina. Arch argent pediatr. 2013;111(4):295–302. 10.5546/aap.2013.eng.295 23912286

[33] 33. Herrera JF, Vargas A, Campos M, Marín JP, Moya T, Herrera ML. Inmunofluorescencia por Bordetella pertussis Hospital Nacional de Niños. Rev Med Hosp Nac Niños 2002;37(1/2):33–9.

[34] 34. Kusznierz G, Schmeling F, Cociglio R, Pierini J, Molina F, Ortellao L, et al. Epidemiologic and clinical characteristics of children with disease due to Bordetella pertussis in Santa Fe, Argentina. Rev Chilena Infectol. 2014;31(4):385–92. 10.4067/S0716-10182014000400002 25327190

[35] 35. Magnabosco Cosner A, Marmitt CR, Rohde LA, Alfonso T, Nery Paes CE. Cobertura e motivos de atrasos vacinais em crianças de Vila Periférica da Grande Porto Alegre. Journal AMRIGS. 1988;32(1):5–8.

[36] 36. Marchese AF. Cobertura de vacunación en tres localidades de la provincia de Santa Fe (1984). Archivos Argentinos de Pediatría. 1985;83(6):322–5.

[37] 37. Merle S, Giboyau J, Vigée D. Couverture vaccinale des enfants en Martinique. Bulletin Épidémiologique Hebdomadaire 2000(19):79–80.

[38] 38. Molina IB, Duron Andino R. La tos-ferina en Honduras. Tegucigalpa: Organización Panamericana de la Salud; 1992.

[39] 39. Monteiro CA, França Júnior I, Conde WL. Evolução da assistência materno-infantil na cidade de São Paulo (1984–1996). Rev Saúde Públ 2000;34(6, Suppl.):19–25. 11428196

[40] 40. Moreno L, Montanaro P, Bujedo E, Camara J, Abilar C, Terzoni M, et al. Predictores de coqueluche al ingreso en lactantes hospitalizados con infección respiratoria aguda baja [Pertussis predictors in hospitalized infants with acute lower respiratory tract infection]. Revista de la Facultad de Ciencias Medicas de Cordoba. 2013;70(2):63–9. 24067589

[41] 41. Morón Duarte LS. Immunopreventable diseases. Public health monitoring of whooping cough in Colombia, 2005 Inf Quinc Epidemiol Nac 2006;11(5):65–80.

[42] 42. Morón Duarte LS, Moreno J, Gracia M, Realpe ME, Peña Daza GL. Estado de portadores de bordetella pertussis en adolescentes de 12 a 19 años en el departamento del Tolima, Colombia, 2007. Investig andin. 2008;10(17):7–26.

[43] 43. Morón-Duarte L, Espetia MT. Evaluación rápida de coberturas vacunales en Bogotá, 2006. Rev Saúde Públ 2009;11(2):237–46. 10.1590/s0124-00642009000200008 19721996

[44] 44. Moura da Silva AA, Gomes UA, Tonial SR, da Silva RA. Cobertura vacinal e fatores de risco associados à näo-vacinaçäo em localidade urbana do Nordeste brasileiro, 1994. Rev Saúde Públ 1999;33(2):147–56. 10.1590/s0034-89101999000200006 10413932

[45] 45. Nieto Guevara J, Luciani K, Montes de Oca Melian A, Mateos Duran M, Estripeaut D. Hospitalizaciones por Bordetella pertussis: experiencia del Hospital del Nino de Panama, periodo 2001–2008. An Pediatr (Barc) [Internet]. 2010 July 1, 2010:1–7. 10.1016/j.anpedi.2009.11.012 20153272

[46] 46. Ochoa-Perez UR, Hernandez-Sierra JF, Escalante-Padron FJ, Contreras-Vidales S, Berman-Puente AM, Hernandez-Maldonado F, et al. Epidemiology of Bordetella pertussis in San Luis Potosi, Mexico. Pediatr Infect Dis J. 2014;33(5):540–2. 10.1097/INF.0000000000000205 24220229

[47] 47. Oliveira e Silva RBd, Lemes-Marques EG, Medeiros MIC, Almeida IAZCd, Esper MRNR, Garbelotti M, et al. Diagnóstico laboratorial da coqueluche: Freqüência do isolamento de Bordetella pertussis de amostras clínicas, por meio da ténica de cultura realizada nos laboratórios regionais do Instituto Adolfo Lutz, São Paulo, Brasil. Rev Inst Adolfo Lutz. 2007;66(2):194–200.

[48] 48. Pan American Health Organization. EPI coverage in Guyana. PAHO EPI Newsletter. 1990;12(5):8.

[49] 49. Perret P C, Vial Claro P, Viviani S T, González A P, Montiel Avendaño F, Riedel K I, et al. Etiología del síndrome coqueluchoídeo y rendimiento de las técnicas para el diagnóstico de bordetella pertussis en pacientes hospitalizados. Rev chil infectol. 1999;16(1):17–26.

[50] 50. Porto LA. Cobertura vacinal nos municípios de Iguaí e Caldeiräo Grande, Bahia, em 1997. Inf Epidemiol Sus. 1998;7(4):7–24.

[51] 51. Quian Rivero JW, Cerisola Cardozo A, Russomano Olivera F, Fernández A, Cappeta M, Uriarte MdR, et al. Infecciones por Bordetella pertussis en niños menores de un año hospitalizados y sus contactos del hogar. Arch pediatr Urug. 2006;77(3):229–36.

[52] 52. Quiri Figueroa OJ, Sabillón Vallecillo N, Almendares Bonilla J. Evaluación del esquema de inmunización en niños menores de un año durante 3 meses de Siguatepeque. Rev Med Hondur. 1991;59(2):79–83.

[53] 53. Rainey JJ, Lacapere F, Danovaro-Holliday MC, Mung K, Magloire R, Kananda G, et al. Vaccination coverage in Haiti: results from the 2009 national survey. Vaccine. 2012;30(9):1746–51. 10.1016/j.vaccine.2011.12.015 22227146

[54] 54. Rios O Jdl, Carvajal P. Jornada departamental de vacunacion, agosto 1 de 1982. Bol epidemiol Antioq. 1982;7(3):75–7.

[55] 55. Riva Posse CA, Miceli INP. Evolución de la coqueluche en la Argentina a finales del siglo XX. Medicina (BAires). 2005;65(1):7–16. 15830787

[56] 56. Rodríguez R, Guerrero R. Evaluación epidemiológica de las primeras jornadas nacionales de vacunación en Colombia, 1984. Colomb Méd. 1985;16(3/4):96–102.

[57] 57. Da Silva LMV. Overestimated vaccination coverage rates? New evidence from the Pau de Lima survey. Rev Panam Salud Publica. 1997;1(6):444–50. 9377653

[58] 58. Szwarcwald CL, Valente JG. Avaliacao da cobertura de vacinação em Teresina - Piauí (Brasil - 1983). Cad Saúde Pública. 1985;1(1):41–9.

[59] 59. Teixeira Antonia Maria da Silva, Domingues Carla Magda Allan S. Monitoramento rápido de coberturas vacinais pós-campanhas de vacinação no Brasil: 2008, 2011 e 2012. Epidemiol serv saúde. 2013;22(4):565–78.

[60] 60. Teixeira de Siqueira M, de Azevedo Mello Romani S. Perfil epidemiológico no município de Olinda, 1990. Rev Bras Saude Mater Infant. 1993;7(1):3–11.

[61] 61. Tomé Sandoval P, Torres Arreola LdP, Romero Quechol G, Guiscafré Gallardo H. Bordetella pertussis en estudiantes adolescentes de la Ciudad de México. Rev Saúde Públ 2008;42(4):679–83. 10.1590/s0034-89102008000400014 18709244

[62] 62. Almada C, Mara P, Perdomo V, Belo J, Salterain Hd, Silva Ed, et al. Whooping cough: outbreak 2011–2012, Hospital Las Piedras. Rev Méd Urug 2015;31(4):265–71.

[63] 63. Berezin EN, Moraes JCd, Leite D, Carvalhanas TRMP, Yu ALF, Blanco RM, et al. Sources of pertussis infection in young babies from Sao Paulo State, Brazil. Pediatr Infect Dis J. 2014;33(12):1289–91. 10.1097/INF.0000000000000424 25386966

[64] 64. Castillo ME, Bada C, Aguila Od, Petrozzi-Helasvuo V, Casabona-Ore V, Reyes I, et al. Detection of Bordetella pertussis using a PCR test in infants younger than one year old hospitalized with whooping cough in five Peruvian hospitals. International Journal of Infectious Diseases. 2015;41:36–41. 10.1016/j.ijid.2015.10.020 26523641

[65] 65. del Valle Mendoza J, Casabona-Ore V, Petrozzi-Helasvuo V, Cornejo-Tapia A, Weilg P, Pons MJ, et al. Bordetella pertussis diagnosis in children under five years of age in the regional hospital of Cajamarca, northern Peru. J Infect Dev Ctries. 2015;9(11):1180–5. 10.3855/jidc.6803 26623626

[66] 66. Guimaraes LM, Carneiro EL, Carvalho-Costa FA. Increasing incidence of pertussis in Brazil: a retrospective study using surveillance data. BMC Infectious Diseases. 2015;15:442. 10.1186/s12879-015-1222-3 PMC4619034 26498058

[67] 67. Pavic-Espinoza I, Bendezu-Medina S, Herrera-Alzamora A, Weilg P, Pons MJ, Aguilar-Luis MA, et al. High prevalence of Bordetella pertussis in children under 5 years old hospitalized with acute respiratory infections in Lima, Peru. BMC Infectious Diseases. 2015;15:554. 10.1186/s12879-015-1287-z PMC4667485 26626910

[68] 68. Perez-Perez GF, Rojas-Mendoza T, Cabrera-Gaytan DA, Grajales-Muniz C. [Pertussis in Mexico, an epidemiological overview. A study of 19 years at the Instituto Mexicano del Seguro Social]. Rev Med Inst Mex Seguro Soc. 2015;53(2):164–70. 25760745

[69] 69. Pimentel AM, Baptista PN, Ximenes RAdA, Rodrigues LC, Magalhães V, Silva ARS, et al. Pertussis may be the cause of prolonged cough in adolescents and adults in the interepidemic period. Braz j infect dis.19(1):43–46. 10.1016/j.bjid.2014.09.001 PMC9427331 25452019

[70] 70. Romanin V, Agustinho V, Califano G, Sagradini S, Aquino A, Del Valle Juarez M, et al. Epidemiological situation of pertussis and strategies to control it. Argentina, 2002–2011. Arch argent pediatr. 2014;112(5):413–20. 10.5546/aap.2014.eng.413 25192521

[71] 71. Santos BAd, Bischoff AR, Chikota C, Silva CC, Lima NBd, Agne M, et al. Coqueluche em crianças menores de 3 anos de idade hospitalizadas em 2011 e 2012. Clin biomed res.35(4):227–32.

[72] 72. Sosa M, Castro M, Salomón S, Giachetto G. Características epidemiológicas y clínicas de niños hospitalizados con tos convulsa durante el 2012 en el Hospital Pediátrico del Centro Hospitalario Pereira Rossell. Arch pediatr Urug. 2014;85(1):10–7.

[73] 73. Ulloa Virgüez AP. Comportamiento epidemiológico de la tos ferina en Colombia. Rev cuba med gen integr. 2015;31(1):42–51.

[74] 74. Torres RS, Santos TZ, Torres RA, Pereira VV, Favero LA, OR MF, et al. Resurgence of pertussis at the age of vaccination: clinical, epidemiological, and molecular aspects. J Pediatr (Rio J). 2015;91(4):333–8. 10.1016/j.jped.2014.09.004 25623040

[75] 75. World Health Organization. WHO-recommended standards for surveillance of selected vaccine-preventable diseases. Geneva: Department of Vaccines and Biologicals, WHO; 2003.

[76] 76. World Health Organization. WHO SAGE pertussis working group. Background paper. 2014 August, 2015. Available from: http://www.who.int/immunization/sage/meetings/2014/april/1_Pertussis_ background_FINAL4_web.pdf?ua=. Accessed in April 2017.

[77] 77. World Health Organization. Pertussis vaccines: WHO position paper. Weekly epidemiological record. 2015;35(90):433–60.

[78] 78. Cherry JD, Grimprel E, Guiso N, Heininger U, Mertsola J, Cherry JD, et al. Defining pertussis epidemiology: clinical, microbiologic and serologic perspectives. Pediatr Infect Dis J. 2005;24(5 Suppl):S25–34. 10.1097/01.inf.0000160926.89577.3b 15876920

[79] 79. Cherry JD, Tan T, Wirsing von König CH, Forsyth KD, Thisyakorn U, Greenberg D, et al. Clinical Definitions of Pertussis: Summary of a Global Pertussis Initiative Roundtable Meeting, February 2011. Clin. Infect. Dis 2012;54(12):1756–64. 10.1093/cid/cis302 PMC3357482 22431797

[80] 80. Faulkner A, Skoff T, Martin S, Cassiday P, Tondella ML, Lian J. Manual for the Surveillance of Vaccine-Preventable Diseases: Pertussis: CDC; 2015. Available from: http://www.cdc.gov/vaccines/pubs/surv-manual/chpt10-pertussis.html. Accessed in May 2016

[81] 81. Van der Zee A, Schellekens J, Mooic F. Laboratory Diagnosis of Pertussis. Clin Microbiol Rev. 2015;28(4):1005–26. 10.1128/CMR.00031-15 PMC4575397 26354823

[82] 82. World Health Organization. Laboratory Manual for the Diagnosis of Whooping Cough caused by Bordetella pertussis/Bordetella parapertussis Update 2014 2014. Available from: http://apps.who.int/iris/bitstream/10665/127891/1/WHO_IVB_14.03_eng.pdf. Accessed in May 2016

[83] 83. World Health Organization, UNICEF. Global Immunization Vision and Strategy 2006–2015. 2005. Available from: http://whqlibdoc.who.int/hq/2005/WHO_IVB_05.05.pdf. Accessed in May 2016 10.2471/BLT.07.045096 PMC2647343 18235887

[84] 84. Kendrick P. Secondary familial attack rates from pertussis in vaccinated and unvaccinated children. Am J Hyg. 1940;32(89–91).

[85] 85. Medical Research Council. The prevention of whooping-cough by vaccination. Br Med J. 1951;2:1463–71. PMC2069253 14839295

[86] 86. Tiwari T, Murphy TV, Moran J. Recommended Antimicrobial Agents for the Treatment and Postexposure Prophylaxis of Pertussis : 2005 CDC Guidelines. MMWR. 2005;54(RR14):1–16. 16340941

[87] 87. Wiley K, Zuo Y, Macartney K, PB M. Sources of pertussis infection in young infants: A review of key evidence informing targeting of the cocoon strategy. Vaccine. 2012. 10.1016/j.vaccine.2012.11.052 23200883

[88] 88. Skoff T, Kenyon C, Cocoros N, Liko J, Miller L, Kudish K, et al. Sources of Infant Pertussis Infection in the United States. J. Pediatr 2015;136(4). 10.1542/peds.2015-1120 26347437

[89] 89. Sizaire V, Garrido-Estepa M, Masa-Calles J, MV MdA. Increase of pertussis incidence in 2010 to 2012 after 12 years of low circulation in Spain. Euro Surveill. 2014;19(32):20875. 10.2807/1560-7917.es2014.19.32.20875 25139074

[90] 90. Skowronski DM, Serres GD, MacDonald D, Wu W, Shaw C, Macnabb J, et al. The Changing Age and Seasonal Profile of Pertussis in Canada. J Infect Dis. 2002;185(10):1448–53. 10.1086/340280 11992280

[91] 91. Cherry JD. Epidemic pertussis in 2012-the resurgence of a vaccine-preventable disease. N Engl J Med. 2012;367(9):785–7. 10.1056/NEJMp1209051 22894554

[92] 92. Shapiro ED. Acellular vaccines and resurgence of pertussis. JAMA. 2012;308(20):2149–50. 10.1001/jama.2012.65031 23188034

[93] 93. Clark TA. Changing Pertussis Epidemiology: Everything Old is New Again. J Infect Dis. 2014;209(7):978–81. 10.1093/infdis/jiu001 24626532

[94] 94. Lavine JS, Bjørnstad ON, de Blasio BF, J. S. Short-lived immunity against pertussis, age-specific routes of transmission, and the utility of a teenage booster vaccine. Vaccine. 2012;30(3):544–51. 10.1016/j.vaccine.2011.11.065 PMC3246080 22119924

[95] 95. Grgic-Vitek M, Klavs I, A K. Re-emergence of pertussis in Slovenia: time to change immunization policy. Vaccine. 2008;26(15):1874–8. 10.1016/j.vaccine.2008.01.045 18313178

[96] 96. PAHO. Vaccination-Your Best Shot: Final Report of Technical Advisory Group on Vaccine-preventable Diseases (TAG) XXII Meeting Washington DC, 1–2 July, 2014.

[97] 97. Althouse BM, Scarpino SV. Asymptomatic transmission and the resurgence of Bordetella pertussis. BMC Med. 2015;13(146). 10.1186/s12916-015-0382-8 PMC4482312 26103968

[98] 98. Gambhir M, Clark TA, Cauchemez S, Tartof SY, Swerdlow L, Ferguson NM. A Change in Vaccine Efficacy and Duration of Protection Explains Recent Rises in Pertussis Incidence in the United States. PLOS Comput Biol. 2015;11(1). 10.1371/journal.pcbi.1004138 PMC4408109 25906150

[99] 99. Ulloa-Gutierrez R, Gentile A, Avila-Aguero ML. Pertussis Cocoon Strategy: Would It Be Useful for Latin America and Other Developing Countries? Expert Rev Vaccines [Internet]. 2012; 11(12):1393–6. Available from: http://www.medscape.com/viewarticle/777379. Accessed in May 2016. 10.1586/erv.12.121 23252382

[100] 100. Advisory Committee on Immunization Practices (ACIP). Updated Recommendations for Use of Tetanus Toxoid, Reduced Diphtheria Toxoid and Acellular Pertussis (Tdap) Vaccine 2010. MMWR. 2010;60(01):13–5. 21228763

[101] 101. Advisory Committee on Immunization Practices (ACIP).. Updated Recommendations for Use of Tetanus Toxoid, Reduced Diphtheria Toxoid, and Acellular Pertussis Vaccine (Tdap) in Pregnant Women 2012. MMWR. 2013;62(07):131–5. PMC4604886 23425962

[102] 102. Vizzottia C, Neyroa S, Katza N, Juáreza MV, Perez Carregaa ME, Aquinoa A, et al. Maternal immunization in Argentina: A storyline from the prospective of a middle income country. Vaccine. 2015. 10.1016/j.vaccine.2015.07.109 26277071

[103] 103. Amirthalingam G, Andrews N, Campbell H, Ribeiro S, Kara E, Donegan K, et al. Effectiveness of maternal pertussis vaccination in England: an observational study. Lancet. 2014;384(9953):1521–8. 10.1016/S0140-6736(14)60686-3 25037990

[104] 104. Dabrera G, Amirthalingam G, Andrews N, Campbell H, Ribeiro S, Kara E, A et al. Case-Control Study to Estimate the Effectiveness of Maternal Pertussis Vaccination in Protecting Newborn Infants in England and Wales, 2012–2013. Clin Infect Dis. 2015;60(3):333–7. 10.1093/cid/ciu821 25332078

